# Proteomic Exploration of L1CAM^+^-Extracellular Vesicles from Plasma of Manifest and Prodromal Parkinson’s Disease

**DOI:** 10.3390/ijms262311564

**Published:** 2025-11-28

**Authors:** Mary Xylaki, Avika Chopra, Yevheniia Kyriachenko, Jannik Scherer, Birgit Otte, Mohammed Dakna, Michael Bartl, Sandrina Weber, Sebastian Schade, Christof Lenz, Brit Mollenhauer

**Affiliations:** 1 Department of Neurology, University Medical Center Göttingen, 37075 Göttingen, Germany; m.xylaki@gmail.com (M.X.); avika.chopra@med.uni-goettingen.de (A.C.); yevheniia.kyriachenko@med.uni-goettingen.de (Y.K.); jannik.scherer@med.uni-goettingen.de (J.S.); birgit.otte@med.uni-goettingen.de (B.O.); mohammed.dakna@med.uni-goettingen.de (M.D.); michael.bartl@med.uni-goettingen.de (M.B.); sandrinakatharina.weber@med.uni-goettingen.de (S.W.); 2 Institute for Neuroimmunology and Multiple Sclerosis Research, University Medical Center Göttingen, 37075 Göttingen, Germany; 3 Paracelsus-Elena Klinik, 34128 Kassel, Germany; 4 Department of Clinical Chemistry, University Medical Center Göttingen, 37075 Göttingen, Germany; christof.lenz@med.uni-goettingen.de; 5 Bioanalytical Mass Spectrometry, Max Planck Institute for Multidisciplinary Sciences, 37077 Göttingen, Germany

**Keywords:** extracellular vesicles, Parkinson’s disease, iRBD, L1CAM, biomarker

## Abstract

L1 cell adhesion molecule (L1CAM)-positive extracellular vesicles (EVs) are being explored as a potential source of biomarkers for Parkinson’s disease (PD) in peripheral blood. However, their utility remains controversial. In this study, we sought to investigate the proteome composition of L1CAM^+^-EVs isolated from human blood plasma and evaluate their potential as biomarkers for PD. L1CAM^+^-EVs were extracted from blood plasma using direct immunoprecipitation by employing magnetic beads coupled to an anti-L1CAM antibody. The Proximity Extension Assay platform, Olink Explore 3072, was used to analyze samples from 60 individuals: 20 healthy controls (HC), 20 patients with isolated REM sleep behavior disorder (iRBD), and 20 PD patients. Targeted proteomic analysis identified 2841 proteins in L1CAM^+^-EVs, of which 203 exhibited differential expression across groups. Although these changes were not statistically significant, after correction for multiple testing, a combination of 12 proteins could discriminate between PD and HC. Moreover, several proteins displayed trends toward upregulation or downregulation in PD and iRBD when compared with HC. These preliminary findings suggest that L1CAM^+^-EVs proteins show some potential as biomarkers for PD; however, further investigation and validation studies are required.

## 1. Introduction

Parkinson’s disease (PD) poses a significant diagnostic challenge due to its complex pathophysiology and the absence of definitive diagnostic tools [1,2], especially in the prodromal phase. Up to 70% of dopaminergic neurons degenerate during this stage, which can occur 20–30 years before the onset of motor symptoms [3]. Therefore, early and accurate diagnostic methods are needed [4], especially as prevention studies continue to evolve.

While post-mortem neuropathological examination of aggregated alpha-synuclein (aSyn) provides a definitive diagnosis, the emergence of aSyn seed amplification assays (SAA) in cerebrospinal fluid (CSF) has now proven effective and accurate for detecting pathological aSyn in isolated REM sleep behavior disorder (iRBD) and in prodromal and early PD stages in vivo [5,6]. This development has led to the conceptualization of an SAA-based NSD-ISS PD staging system. However, this system fails to accurately represent disease progression because it relies on largely static biomarkers—such as aSyn-SAA status, SNCA genotype, and binary DAT-SPECT measures. Since the aSyn-SAA only predicts the presence or absence of aSyn seeds in a patient sample, it does not capture dynamic biological changes, resulting in inappropriate stage distributions, apparent “stage regression” under symptomatic treatment [7], and a lack of correlation with established neurodegeneration biomarkers. Therefore, it remains necessary to identify biomarkers that can predict disease progression before the onset of motor symptoms, ideally ones that capture dynamic molecular and cellular changes, that more accurately reflect the underlying pathophysiology and temporal evolution of PD.

Efforts in biomarker research have focused on various sample sources, including CSF and peripheral biological specimens. However, the invasiveness of CSF collection and the need for a trained medical professional limit its clinical application. Extracellular vesicles (EVs) have emerged as promising candidates due to their role in intercellular communication and their presence in easily accessible biofluids [8], like peripheral blood. EVs are secreted by all cell types in health and disease. They are enclosed by a bilipid layer and carry cargoes including proteins, glycolipids, and nucleic acids, and contain several proteins making up the corona [9,10]. The surface proteins can categorize the vesicles based on their cellular origin [11,12]. Many studies use the surface marker L1CAM for immunoprecipitation (IP) to enrich neuronal-origin EVs from blood [13,14,15], although the exact nature, origin, and nomenclature of these isolates continue to be debated [16]. Nevertheless, previous studies have demonstrated that blood-derived EVs displaying L1CAM ectodomain epitopes also carry neuronal proteins whose abundance increases with L1CAM^+^ EV enrichment. Moreover, soluble L1CAM does not associate with the EV corona, thereby suggesting L1CAM as a suitable target for isolating neuron-derived EVs from blood [17]. Consistent with these findings, our recent review and meta-analysis demonstrated that L1CAM-IP EVs reliably distinguish PD patients from healthy controls (HC) [18]. To date, most studies have been hypothesis-driven, focusing on assessing proteins known to contribute to disease development [19,20,21]. In this exploratory pilot study, we investigated whether L1CAM^+^-EVs can be employed to find markers for diagnostic and prognostic purposes in the clinical spectrum of prodromal to manifest PD. For this, we performed L1CAM-IP on blood plasma to isolate EVs and assess randomly selected iRBD, PD, and HC participants from our cohorts with the exploratory panel from Olink covering 3072 plasma proteins [22].

## 2. Results

### 2.1. Demographics

The study cohort consisted of three groups: 20 PD patients (79% male), 20 participants with iRBD (55% male), and 20 HCs (75% male). The mean ages were 65 ± 11 years for the PD group, 67 ± 9 years for the iRBD group, and 64 ± 8 years for the HC group. The average motor score, assessed using the Unified Parkinson’s Disease Rating Scale (UPDRS) Part III, was 18 ± 7 in the PD group (in the HC and iRBD groups, motor score was assessed using the Movement Disorders Society (MDS)-UPDRS scale). Cognitive performance, assessed using the Mini-Mental State Examination (MMSE), did not differ significantly across groups, with all groups demonstrating a comparable mean score of 28 (Table 1).

### 2.2. Characterization of L1CAM^+^-EVs Isolated from Plasma

**Figure 1:**
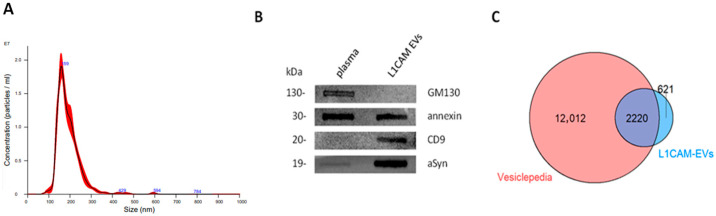
**Characterization of L1CAM^+^-EVs and their cargo.** (**A**). Quantification of L1CAM-EVs concentration and size by NTA. (**B**). Immunoblotting analysis for negative and positive EV markers. (**C**). Venn diagram showing the overlap of L1CAM-EV cargo with the EV cargo database published in Vesiclepedia.

The EVs isolated via IP were characterized using nanoparticle tracking analysis (NTA), and our results were consistent with other studies [14] where the isolated EVs exhibited a particle size ranging between 100 and 300 nm, with the peak at 168 ± 60 nm and a concentration of 2.74 × 10^9^ ± 0.15 × 10^9^ particles/mL (Figure 1A). Immunoblotting analysis validated the purity of EVs by the enrichment of EV-marker CD9 and the absence of GM130, which indicates the absence of contamination with cell debris, apoptotic bodies, or organellar components, such as from Golgi-derived membranes (Figure 1B). aSyn was also enriched in the L1CAM^+^-EVs when compared with total plasma (Figure 1B). The eluate from the negative IP controls did not display any protein bands on the slot blot, confirming the absence of non-specific EV binding.

### 2.3. Biomarker Panel Results

Using the Olink Explore 3072 panel, we detected and quantified a total of 2841 proteins within the L1CAM^+^-EVs. Comparison with the Vesiclepedia database [23] demonstrated that 84.4% of the proteins detected in our study have been previously reported in EVs, supporting the robustness of the L1CAM IP-based EV isolation method (Figure 1C).

Among the quantified proteins, 203 initially showed a significantly differential expression across the PD, iRBD, and HC groups (raw *p*-value < 0.05 in ANOVA analysis); although these differences were rendered insignificant after FDR multiple testing corrections using the Benjamini–Hochberg procedure at level 0.05. The underlying reason is likely the small sample size in this pilot study and the very high number of simultaneously tested proteins (3072).

**Figure 2:**
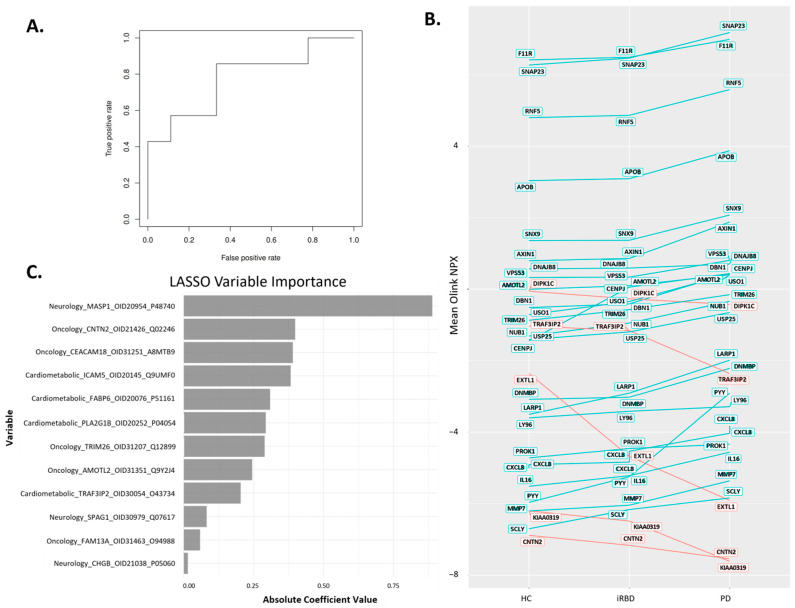
**Differentiation of PD, iRBD, and HC based on markers predicted using the LASSO classifier model.** (**A**). ROC curve analysis for differentiating PD vs. HC (AUC = 0.77) (**B**). The expression of 25 proteins was observed to be downregulated (represented in blue) while five proteins were upregulated (represented in red) when HC were compared with iRBD and subsequently with PD. (**C**). Variable importance plot from the LASSO regression model, highlighting the proteins and their contribution to the classification of PD versus HC. The top predictors included MASP1, CNTN2, CEACAM18, ICAM5, and FABP6. Bars represent the absolute values of the LASSO coefficients, indicating each protein’s relative influence on the model’s discriminative power.

The logistic regression with LASSO variable selection showed a subset of twelve different proteins that could discriminate PD from HC (*p* = 0.0061) with an area under the curve of 0.77 (Figure 2A). The threshold at the Youden index (J = sens + spec-1) is 0.478 with a sens = 0.857 and spec = 0.667. Among those twelve proteins, FABP6, ICAM5, PLA2G1B, TRAF3IP2 were from the cardiometabolic panel; CHGB, MASP1, SPAG1 from the neurology panel; and AMOTL2, CEACAM18, CNTN2, FAM13A, TRIM26 from the oncology panel (Supplementary Table S3). Interestingly, out of the 20 iRBD participants, our classifier model categorized 14 as PD and 6 as HC.

Moreover, among the 203 proteins that were nominally significant prior to multiple testing correction, 30 proteins exhibited a monotonic trend across disease progression, with 25 showing a stepwise increase and five showing a stepwise decrease in their mean abundance from HC → iRBD → PD (Figure 2B, Supplementary Tables S4 and S5). The added value of these 30 proteins, which describe the pathophysiology of PD progression, must be assessed in a large cohort, as none of them reached statistical significance after multiple testing adjustments in our present study. LASSO regression model highlighting the proteins and their contribution to the classification of PD versus controls suggested that MASP1, CNTN2, CEACAM18, and ICAM5 had the highest prediction (Figure 2C).

**Figure 3:**
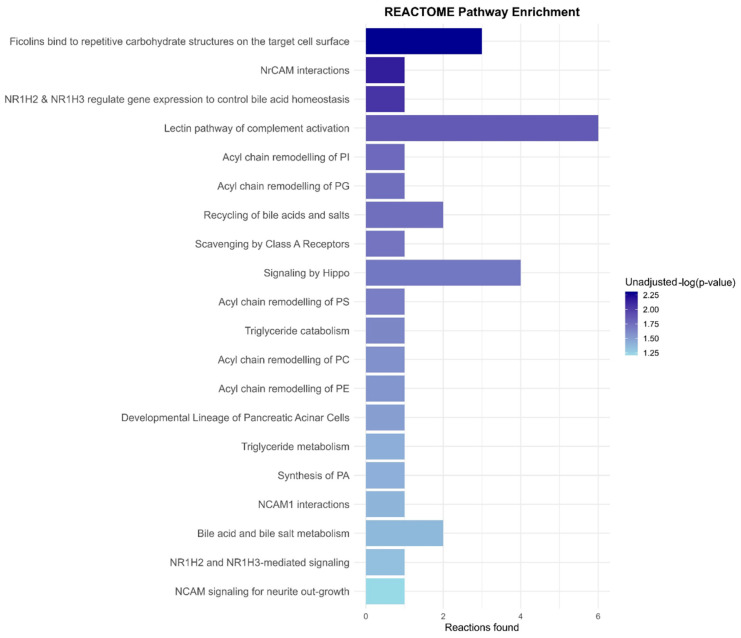
**The Reactome pathway enrichment analysis of pathways mediated by the 12 differentially regulated proteins.** In total, 20 statistically significant pathways are shown, and their respective −log(*p*-values) are indicated alongside the bars with a color-based legend.

Further, to assess which pathways could be potentially influenced, a Reactome analysis was performed for the 12 proteins that were differentially regulated in PD vs. HC (Supplementary Table S6). We observed the emergence of pathways including NCAM signaling for neurite out-growth, NR1H2 and NR1H3 mediated signaling pathways, and bile acid salt metabolism, among others (Figure 3).

## 3. Discussion

In this study, we explored L1CAM^+^-EVs isolated from plasma as a source of potential biomarkers that can be easily obtained and enable differential diagnosis between PD, iRBD, and HC. We quantified 2841 proteins from the L1CAM^+^-EVs; of these, 203 exhibited potential differential expression across the groups.

Among the proteins quantified from L1CAM^+^-EVs using the Olink Explore panel, 84.4% overlapped with previously reported EV proteins in the Vesiclepedia database (http://microvesicles.org/, accessed on 6 October 2025). The comparison with our previous total plasma proteomics study using Olink [24] found no overlap between proteins inside L1CAM^+^-EVs and total plasma proteomics, indicating distinct proteomic profiles. These findings suggest that the L1CAM-IP provides a potential method for EV isolation and subsequent protein profiling with minimal plasma contamination. However, further investigation is necessary to assess the purity of samples for future experiments.

A panel of twelve proteins (FABP6, ICAM5, PLA2G1B, TRAF3IP2, CHGB, MASP1, SPAG1, AMOTL2, CEACAM18, CNTN2, FAM13A, and TRIM26) effectively differentiated between PD and HC with a reported AUC of 0.77.

Intercellular adhesion molecule 5 (ICAM-5) plays a dual role in the CNS, exerting neuroprotective effects in progressive neurodegeneration [25] and modulating microglial activation by reducing the secretion of proinflammatory factors and inducing the release of anti-inflammatory factors [26]. Reduced levels of its secreted form have been observed in the CSF of patients with multiple sclerosis [25], while elevated concentrations in blood plasma have been associated with acute neuronal injury, as seen during the first 24 h after traumatic brain injury [27]. Thus, increased plasma levels of ICAM-5 found in our study may reflect enhanced neuronal death and subsequent release of this membrane-bound adhesion molecule. Chromogranin B (CHGB) is a key component of the regulated secretory pathway, involved in the formation and function of dense-core vesicles that store and release neurotransmitters such as dopamine [28,29]. It has been consistently reported to show reduced levels in the CSF of patients with PD [30,31]. Reduced CHGB levels may reflect impaired vesicle transport and secretion, contributing to the catecholaminergic deficit and neuronal dysfunction in PD progression. This is in line with our previous findings showing that neurosecretory protein VGF, another member of the granin family, is decreased in CSF of PD and iRBD individuals [32]. However, we found that CHGB is upregulated in L1CAM^+^-EVs of PD patients, suggesting a shift in its secretion dynamics. In PD, disruption of the regulated secretory pathway may lead to CHGB being misdirected into exosomes or microvesicles instead of dense-core vesicles, with stressed neurons potentially enhancing CHGB-containing EV release as a compensatory response. Angiomotin-like protein 2 (AMOTL2) is a key regulator within the Hippo signaling pathway, where it modulates neural stem cell proliferation and differentiation by repressing YAP activity [33]. AMOTL2 undergoes phosphorylation via the mTORC2-mediated pathway, thereby enhancing YAP signaling, which could disrupt neurogenesis and contribute to mechanisms underlying PD [34,35]. The elevated AMOTL2 levels observed in our study may, therefore, reflect an adaptive, neuroprotective response aimed at promoting neural stem cell activation and compensating for dopaminergic neuron loss during PD progression. Contactin-2 (CNTN2) is a neuronal cell adhesion molecule essential for axonal conduction, myelination, and synaptic integrity [36]. A recent study reported no significant changes in CSF CNTN2 levels in PD; however, the authors observed a clear reduction in contactin-1 and identified both contactins within Lewy bodies and neurites, linking them to aSyn–related synaptic degeneration [37]. In our study, we observed decreased CNTN2 levels in PD patients, suggesting a potential role of CNTN2 in synaptic dysfunction in PD progression. Moreover, CNTN2 marks dopaminergic progenitors capable of functional dopamine release after transplantation, suggesting that, beyond its role in maintaining synaptic connectivity, CNTN2 could also contribute to regenerative mechanisms that hold relevance to treatments for PD [38]. In previous publications, we showed that Contactin 4 levels are also decreased in CSF in PD and that Contactin 1 CSF levels are significantly correlated with changes in non-motor and PD progression scores over 8 years [39].

Fatty acid binding protein 6 (FABP6), a key transporter responsible for bile acid reuptake in the ileum, is markedly reduced in the gut of PD patients, indicating impaired enterohepatic circulation of bile acids [40]. This disruption may contribute to increased levels of proinflammatory secondary bile acids, altered lipid metabolism, and gut dysbiosis, which are factors that could promote aSyn aggregation and gut-to-brain propagation of PD pathology [41,42]. Plasma levels of fatty acid binding protein 4 (FABP4), a member of the same protein family, showed a positive correlation with the MDS-UPDRS III in a previous analysis, suggesting that higher levels are associated with worse motor symptoms [24]. We observed an increase in FABP6 levels in the blood plasma of PD patients vs. HCs, which could indicate worse motor symptoms. PLA2G1B is a secreted group 1B phospholipase A2 that hydrolyzes membrane phospholipids and has been implicated in metabolic disorders (obesity/diabetes, hyperlipidemia, atherosclerosis), but it has not yet been linked to PD [43]. In our study, we observed that the concentration of this protein was elevated in PD patients, which might indicate the dysregulation of metabolic processes. In contrast, another family member—lipoprotein-associated phospholipase A2 (Lp-PLA2) — has been found to be elevated in the blood of PD and AD patients. It has been strongly associated with cognitive decline, vascular inflammation, and blood–brain barrier (BBB) dysfunction, suggesting that PLA2-mediated lipid signaling could be relevant to PD [44,45,46]. The role of PLA2G1B in regulating neurovascular or lipid-metabolic pathways in PD remains unclear and requires further investigation.

TRAF3 interacting protein 2 (TRAF3IP2) is an adaptor protein regulating NF-κB signaling downstream of the IL-17 pathway and is known to promote proinflammatory and proangiogenic signaling in glioblastoma and breast cancer [47,48]. Although it has not previously been associated with PD, we observed a reduction in TRAF3IP2 levels in L1CAM^+^-EVs from PD patients. Given that silencing TRAF3IP2 lowers the expression of proinflammatory cytokines (IL-1β, IL-6, IL-8) and angiogenic mediators [49], its downregulation in neuronal EVs may indicate a compensatory mechanism aimed at decreasing neuroinflammation or limiting inflammatory signaling in PD pathology. Mannan-Binding Lectin Serine Protease 1 (MASP1), a serine protease of the lectin complement pathway, can activate endothelial cells and trigger proinflammatory signaling via MAPK, NF-κB, and protease-activated receptor pathways, leading to IL-6 and IL-8 release [50,51]. Although its role in PD has not been directly established, the observed downregulation of related complement components, such as MASP2 and its inhibitor SERPING1, in the plasma of iRBD and PD patients has been observed. In our study, MASP1 was also downregulated in EVs, which could contribute to neuroinflammation and alter vascular processes underlying PD pathology. Further, these markers were suitable for distinguishing PD patients from HCs and could help diagnose a phenoconversion from iRBD to motor PD years before motor symptom onset [52]. Sperm associated antigen 1 (SPAG1) is involved in the assembly of ciliary dynein arms, and mutations in its gene are known to cause primary ciliary dyskinesia, a disorder characterized by defective motile cilia and impaired cellular transport [53]. Although SPAG1 has not yet been linked to PD, elevated plasma levels have been associated with faster memory decline in AD, suggesting dysfunctional cilia and a dysregulation of intracellular transport mechanisms, which may contribute more broadly to neurodegenerative processes [54]. In our study, we observed that the levels of this protein were reduced in EVs from PD patients, which could suggest dysregulation of the intracellular transport mechanism and accumulation of toxic proteins in the cells.

The specific biological function of carcinoembryonic antigen-related cell adhesion molecule 18 (CEACAM18) remains poorly understood, and its role in PD has not yet been investigated. As a member of the CEACAM family, CEACAM18 is believed to participate in cell adhesion, immune modulation, and cell–cell communication [55]. Other CEACAMs, such as CEACAM1, have been studied more extensively and shown to regulate inflammatory responses and protect the BBB by inhibiting neutrophil-mediated tissue damage following ischemic injury [56]. Interestingly, elevated serum carcinoembryonic antigen (CEA) levels have been reported in PD patients, which could suggest that the dysregulation of CEACAM family members might influence the inflammatory state of PD [57]. Given these links, CEACAM18 may represent an unexplored modulator of neuroinflammation or BBB integrity in PD and warrants further investigation as a potential biomarker or immune-regulatory factor in neurodegeneration. Our observations align with previously published data, as we also saw an increase in CEACAM18 levels in EVs.

The family with sequence similarity 13 member A (FAM13A) protein encodes a signaling regulator involved in GTPase-mediated pathways and is highly expressed in excitatory neurons, including those of the substantia nigra [58]. Although it has only recently been considered in the context of PD, it lies within the *SNCA* genomic region, a key locus in PD pathogenesis [58]. We observed an upregulation in the levels of FAM13A in EVs from PD patients, which might indicate its contribution to neuronal vulnerability and disease progression through disrupted signaling and potential interaction with aSyn-related pathways. TRIM26 is an E3 ubiquitin ligase of the tripartite motif (TRIM) family, involved in protein ubiquitination, oxidative stress responses, and regulation of DNA repair enzymes such as NEIL1, NEIL3, and OGG1 under conditions of cellular stress [59]. Members of the TRIM family, including TRIM11, TRIM25, and TRIM28, are increasingly recognized for their roles in maintaining proteostasis and preventing aggregation of misfolded proteins such as aSyn [60]. In our study, TRIM26 was upregulated in L1CAM^+^-EVs from PD patients, which may indicate a compensatory neuronal response aimed at counteracting oxidative stress and ferroptosis or enhancing protein quality control through ubiquitin-mediated pathways [61,62]. Based on the functions of these proteins, homeostatic pathways such as inflammation, lipid metabolism, and cellular transport, which are known to be impacted in PD, were identified, although further validation is needed to confirm these results.

Classification of the 20 iRBD patients by our LASSO regression model resulted in 14 being assigned to the PD group and 6 to the HC group, indicating that the proteomic profiles of iRBD patients more closely align with those of individuals with PD than with HC. However, because the model was derived from cross-sectional data and not designed to distinguish PD from iRBD directly, these assignments should be interpreted as reflecting molecular similarity rather than diagnostic prediction.

Although the differential analysis did not retain statistical significance after correction for multiple testing, the observation that 30 out of 203 nominally significant proteins displayed a monotonic trend across the HC → iRBD → PD continuum suggests potential molecular alterations accompanying disease progression and has to be confirmed in a larger cohort. Specifically, 25 proteins showed a gradual upregulation while five proteins showed a consistent decrease in mean abundance, indicating changes that may emerge as early as the prodromal stage and later affect the disease progression. While preliminary, these trends could reflect early pathophysiological mechanisms that require further investigation. Additionally, longitudinal studies are needed to confirm the robustness and clinical relevance of these trends.

Moreover, aSyn was enriched in EVs isolated with L1CAM-IP from HC samples in comparison to the total plasma. This observation aligns with previous studies reporting aSyn enrichment within L1CAM^+^-EVs [18]. Collectively, these findings support L1CAM-based immunocapture as a valuable platform for protein biomarker discovery and underscore the potential of L1CAM^+^-EV cargo as a sensitive molecular indicator of PD pathology.

We recently demonstrated the power of mass spectrometry-based blood proteomics in predicting PD up to seven years before the onset of motor symptoms using a specific blood panel [52], and L1CAM^+^-EVs may hold additional diagnostic value. Platforms, such as Olink, offer many advantages, like high throughput via sample multiplexing and higher sensitivity. These advantages are particularly relevant as the field moves towards the reliable identification of individuals at-risk or population-based cohorts for prevention trials. Although CSF SAA has yielded high accuracy in identifying prodromal patients [63,64], the invasiveness of lumbar punctures renders CSF unsuitable for large-scale screening. Additionally, CSF aSyn SAA are not yet quantitative, nor can they be used as progression markers; SAA in peripheral blood needs further optimization for a robust analysis [65]. Therefore, an alternative blood-based biomarker detection method like ours could benefit patients and medical professionals by providing fast and reliable results, as blood acquisition is less invasive and easily accessible.

In conclusion, our study demonstrates the feasibility of using L1CAM^+^-EVs, isolated from plasma, for protein biomarker discovery in PD and iRBD. Further validation studies with larger cohorts and longitudinal assessments are necessary to clarify the potential of these EV-based biomarkers for monitoring disease progression and evaluating therapeutic interventions in neurodegenerative diseases like PD, a project that we are already working on.

## 4. Materials and Methods

Participants were selected randomly and included age- and sex-matched individuals with PD who are enrolled in the Kassel cohort-I and individuals with iRBD, and HCs enrolled in the de novo Parkinson’s disease (DeNoPa) cohort. Inclusion and exclusion criteria have been described previously [66,67]. Deep clinical phenotyping included motor function as assessed by the UPDRS Part III [68] and MDS-UPDRS Part III, and cognitive function by the MMSE [69]. iRBD was diagnosed with video polysomnography [70].

### 4.1. Sample Collection

EDTA blood plasma samples were collected in the morning under fasting conditions following strict standard operating procedures (SOPs) as previously described [66]. Briefly, 8–10 mL of venous blood was drawn into BD Vacutainer^®^ tubes containing ethylenediaminetetraacetic acid (EDTA) and gently inverted to mix. Within 30 min of collection, samples were centrifuged at 2500× *g* for 10 min at 20 °C. The resulting plasma was carefully aliquoted and stored at −80 °C until further analysis.

### 4.2. Immunoprecipitation

L1CAM^+^-EVs were isolated from plasma by IP using an anti-L1CAM antibody (ab208155, clone number EPR18750, Abcam, Cambridge, Cambridgeshire, UK) and Dynabeads™ Protein G magnetic beads (10004D, Invitrogen, Carlsbad, CA, USA), as per the manufacturer’s protocol with minor modifications. In short, 50 µL magnetic beads were incubated with 6 µg antibody in 200 µL phosphate-buffered saline (PBS), pH 7.4, with 0.02% Tween-20 for 2 h at room temperature with rotation on the carousel. After incubation, the antibody-buffer mixture was removed, and 0.5 mL plasma was mixed with the bead-Ab complex, followed by an overnight incubation at 4 °C with rotation. The following day, plasma was removed, and the beads were washed three times with 200 µL PBS. The bead-PBS suspension was transferred to a clean tube, and after the final wash, beads were resuspended in 20 µL elution buffer (50 mM glycine pH 2.8) or 20 µL 1× RIPA buffer (20-188, Millipore, Burlington, MA, USA) supplemented with 1 mM phenylmethylsulfonyl fluoride (PMSF, P7626, Sigma-Aldrich, St. Louis, MO, USA), and incubated for 20 min at room temperature under vigorous shaking. Negative controls to check for the non-specific binding were performed with only beads or isotype control-coated beads. The supernatant was collected and stored at −80 °C after protein quantification with the Pierce protein assay (22660, Thermo Scientific, Waltham, MA, USA) using the LVis microplate on an Omega Fluorometer (FLUOstar Omega Microplate reader, MARS version: 4.00 R2, BMG LABTECH, Ortenberg, Baden-Württemberg, Germany).

### 4.3. Nanoparticle Tracking Analysis (NTA)

NTA was performed on freshly eluted EVs on NanoSight NS500 LM10 with an LM14 viewing unit equipped with a 532 nm laser (NanoSight Ltd., Amesbury, Wiltshire, UK). Data was recorded using NTA 2.3 software with a detection threshold of 5, captured with a camera level of 16 at 22 °C. Five consecutive 60-s videos and measurements were recorded and used for analysis.

### 4.4. Immunoblotting Analysis

EV lysates were mixed with 5x Laemmli buffer (250 mM Tris pH 6.8, 10% SDS, 1.25% Bromophenol Blue, 5% β-mercaptoethanol, 50% glycerol) and incubated at 95 °C for 5 min. Electrophoresis was performed on Mini-Protean TGX 12% gels (4561044, Bio Rad, Hercules, CA, USA), and proteins were transferred onto the 0.45 µm nitrocellulose membrane (162-0117, Bio Rad).

Immunoblotting (slot-blot) was performed by loading 10 µg of samples from HC with the following antibodies: anti-Syn1, mouse (610786 BD); anti-GM130, rabbit (12480 Cell Signaling); anti-Annexin V, rabbit (8555 Cell Signaling); anti-CD9, rabbit (13174 Cell Signaling). Secondary detection was performed with HRP-conjugated antibodies against mouse (7076P2 Cell Signaling) and rabbit (7074P2 Cell Signaling). Visualization was performed with Fusion FX, Vilber Lourmat.

### 4.5. Biomarker Panel Analysis

We analyzed proteins using the Olink Explore 3072 platform, which employs proximity extension assays where pairs of oligonucleotide-labeled antibodies bind to target proteins, allowing hybridization and subsequent quantification via next-generation sequencing [71]. The platform includes eight panels, each containing 384 assays that target specific biological processes: Cardiometabolic I-II, Inflammation I-II, Neurology I-II, and Oncology I-II [https://olink.com/products/olink-explore-3072-384, accessed on 5 December 2023].

### 4.6. Statistical Analysis

Statistical analyses were performed with R (version 4.3.2; R Core Team 2018). The significance level was set to alpha = 5% for all tests. Group comparisons of continuous variables were made with the non-parametric Kruskal–Wallis test because of some non-normal distributions (UPDRS-III and MMSE). The Freeman–Halton extension of Fisher’s exact test was used for comparing the nominal variable sex with two categories over three groups (HC, iRBD, PD). To identify the most predictive proteins for the HC-PD discrimination while controlling for overfitting, we employed least absolute shrinkage and selection operator (LASSO) logistic regression using the R package (version 4.5.2) glmnet (CRAN). The LASSO approach applies L1 regularization, which shrinks coefficients toward zero and performs automatic feature selection by setting coefficients of non-informative variables to exactly zero. The LASSO logistic regression model was specified as logit(P(Y = 1|X)) = β_0_ + ∑βj Xj, where Y is the binary outcome variable (0 = HC, 1 = PD). X represents the OLINK expression matrix with the NPX values of the biomarker/protein candidates, β_0_ is the intercept term, and βj are the coefficients subject to regularization. We computed the regularization path over 100 λ values (the tuning parameter controlling shrinkage) using 10-fold cross-validation to determine the optimal. The optimal (minimal) λ that minimizes the binomial deviance was 0.085. This resulted in the selection of 12 proteins with non-zero coefficients at the optimal λ. We examined potential multicollinearity among selected proteins using variance inflation factors (VIF). In contrast to conventional stepwise selection methods, the LASSO statistical approach allowed us to identify a set of biomarkers with reasonable performance while minimizing overfitting to the training data. We assessed the diagnostic performance of our biomarker panel using receiver operating characteristic (ROC) analysis with the R package pROC (CRAN), which was also used for computing AUC and performing DeLong’s test to calculate the corresponding *p*-values.

Some of the proteins quantified by the Olink platform are highly correlated, as this method targets many proteins from the same pathway. This can lead to collinearity issues that may cause problems in regression/classification models, depending on their sensitivity to this issue. As reported in the supplement, we compared the LASSO classifier to two other classifiers, including the RIDGE regression, by setting (alpha = 0) in *glmnet* and ElasticNet Regression (alpha = 0.5) (for LASSO alpha = 1) (Supplementary Table S1, Supplementary Figure S1). The analysis shows that for the actual data with a rather small sample size, the LASSO provides a good compromise between model complexity and performance (Supplementary Table S2, Supplementary Figure S2).

EV cargo proteins data were obtained from the Vesiclepedia 2024 [23] by filtering for proteins and Homo Sapiens.

### 4.7. Data Sharing

The data that support the findings of this study are not publicly available. However, the data and related analysis scripts are available from the corresponding author upon reasonable request. Access to the data will be provided in compliance with institutional and ethical guidelines.

## Figures and Tables

**Table 1:** Demographics of the subgroups.

	HC (N = 20)	iRBD (N = 20)	PD (N = 20)	*p* Value
**SEX**				0.377
Female	5 (25.0%)	9 (45.0%)	6 (30.0%)	
Male	15 (75.0%)	11 (55.0%)	14 (70.0%)	
Missing	0	0	0	
**AGE**				0.598
Mean (SD)	64.850 (7.680)	67.100 (9.188)	65.000 (11.007)	
Median (Q1, Q3)	67.000 (63.750, 69.000)	69.500 (62.000, 74.000)	67.500 (54.500, 72.000)	
Min–Max	44.000–77.000	50.000–80.000	46.000–85.000	
Missing	0	0	0	
**MDS_UPDRS_III**				
Mean (SD)	0.200 (0.894)	1.750 (2.099)	NA	
Median (Q1, Q3)	0.000 (0.000, 0.000)	1.000 (0.000, 2.250)	NA	
Min–Max	0.000–4.000	0.000–7.000	NA	
Missing	0	0	20	
**MDS_UPDRS_Total Score**				
Mean (SD)	2.950 (2.685)	15.750 (8.065)	NA	
Median (Q1, Q3)	2.000 (0.750, 5.250)	15.000 (12.000, 17.250)	NA	
Min–Max	0.000–8.000	2.000–35.000	NA	
Missing	0	0	20	
**UPDRS_III**				
Mean (SD)	NA	NA	18.000 (7.189)	
Median (Q1, Q3)	NA	NA	17.000 (14.750, 19.500)	
Min–Max	NA	NA	6.000–35.000	
Missing	20	20	0	
**UPDRS_Total Score**				
Mean (SD)	NA	NA	29.400 (13.697)	
Median (Q1, Q3)	NA	NA	26.000 (20.500, 37.750)	
Min–Max	NA	NA	12.000–61.000	
Missing	20	20	0	
**MMSE_Total_Score**				0.989
Mean (SD)	28.600 (0.940)	28.450 (1.504)	28.071 (2.433)	
Median (Q1, Q3)	29.000 (28.000, 29.000)	29.000 (28.000, 29.250)	28.500 (27.250, 30.000)	
Min–Max	27.000–30.000	24.000–30.000	21.000–30.000	
Missing	0	0	6	

Abbreviations: HC: healthy control; iRBD: isolated REM sleep behavior disorder; PD: Parkinson’s disease; SD: standard deviation; MDS-UPDRS: Movement Disorder Society-Unified Parkinson’s Disease Rating Scale; MMSE: Mini-Mental-State-Examination.

## Data Availability

Raw data supporting the conclusions of this article will be made available by the authors on request.

## Supplementary Materials

The following supporting information can be downloaded at: https://www.mdpi.com/article/10.3390/ijms262311564/s1.

## Abbreviations

The following abbreviations are used in this manuscript:

AMOTL2	Angiomotin Like 2
aSyn	Alpha-Synuclein
AUC	Area Under Curve
CEACAM18	Carcinoembryonic antigen-related cell adhesion molecule 18
CHGB	Chromogranin B
CNTN2	Contactin 2
CSF	Cerebrospinal fluid
DeNoPa	de novo Parkinson’s disease
EDTA	Ethylenediaminetetraacetic acid
EVs	Extracellular vesicles
FABP6	Fatty acid binding protein 6
FAM13A	Family With Sequence Similarity 13 Member A
GM130	Golgi matrix protein 130
HCs	Healthy controls
ICAM5	Intercellular adhesion molecule 5
IP	Immunoprecipitation
iRBD	Isolated REM sleep behavior disorder
L1CAM	L1 cell adhesion molecule
MASP1	Mannan-binding lectin serine protease 1
MMSE	Mini-mental state examination
NTA	Nanoparticle tracking analysis
PA	Phosphatidic acid
PC	Phosphatidylcholine
PD	Parkinson’s disease
PE	Phosphatidylethanolamine
PG	Phosphatidylglycerol
PI	Phosphatidylinositol
PLA2G1B	Phospholipase A2 Group IB
PMSF	Phenylmethylsulfonyl fluoride
PS	Phosphatidylserine
ROC	Receiver operating characteristic
SAA	Seed amplification assay
SPAG1	Sperm associated antigen 1
TGX	Tris-Glycine eXtended
TRAF3IP2	TRAF3 Interacting Protein 2
TRIM26	Tripartite Motif Containing 26
UPDRS	Unified Parkinson’s disease rating scale
