# Supplementary material for: Proteomic Exploration of L1CAM+-Extracellular Vesicles from Plasma of Manifest and Prodromal Parkinson’s Disease
Source: Int J Mol Sci. 2025 Nov 28;26(23):11564. doi: 10.3390/ijms262311564 (PMC12692637; doi:10.3390/ijms262311564)
Supplement: Supplementary file 1 [file ijms-26-11564-s001.zip › Supplementary Table S6.pdf]

	Entities	Entities	Entities	Entities	Entities	Reactions	Reactions	Reactions	
**Pathway name**	**found**	**Total**	**ratio**	**pValue**	**FDR**	**found**	**total**	**ratio**	**Species name**
Ficolins bind to repetitive carbohydrate structures on the target cell surface	1	5	0	4.95E-3	1.14E-1	*3*	*3*	*0*	Homo sapiens
NrCAM interactions	1	7	0.001	6.93E-3	1.14E-1	*1*	*4*	*0*	Homo sapiens
NR1H2 & NR1H3 regulate gene expression to control bile acid homeostasis	1	9	0.001	8.9E-3	1.14E-1	*1*	*6*	*0*	Homo sapiens
Lectin pathway of complement activation	1	14	0.001	1.38E-2	1.14E-1	6	*6*	*0*	Homo sapiens
Acyl chain remodelling of PI	1	17	0.001	1.68E-2	1.14E-1	*1*	*6*	*0*	Homo sapiens
Recycling of bile acids and salts	1	18	0.001	1.77E-2	1.14E-1	*2*	*17*	*0.001*	Homo sapiens
Acyl chain remodelling of PG	1	18	0.001	1.77E-2	1.14E-1	*1*	*10*	*0.001*	Homo sapiens
Scavenging by Class A Receptors	1	19	0.002	1.87E-2	1.14E-1	*1*	*10*	*0.001*	Homo sapiens
Signaling by Hippo	1	20	0.002	1.97E-2	1.14E-1	*4*	*30*	*0.002*	Homo sapiens
Acyl chain remodelling of PS	1	22	0.002	2.16E-2	1.14E-1	*1*	*8*	*0.001*	Homo sapiens
Triglyceride catabolism	1	24	0.002	2.36E-2	1.14E-1	*1*	*17*	*0.001*	Homo sapiens
Acyl chain remodelling of PC	1	27	0.002	2.65E-2	1.14E-1	*1*	*9*	*0.001*	Homo sapiens
Acyl chain remodelling of PE	1	29	0.002	2.84E-2	1.14E-1	*1*	*9*	*0.001*	Homo sapiens

Developmental Lineage of Pancreatic Acinar Cells	1	32	0.003	3.13E-2	1.14E-1	*1*	*3*	*0*	Homo sapiens
Triglyceride metabolism	1	38	0.003	3.71E-2	1.14E-1	*1*	*24*	*0.002*	Homo sapiens
Synthesis of PA	1	39	0.003	3.81E-2	1.14E-1	*1*	*17*	*0.001*	Homo sapiens
NCAM1 interactions	1	42	0.003	4.09E-2	1.23E-1	*1*	*10*	*0.001*	Homo sapiens
Bile acid and bile salt metabolism	1	44	0.004	4.28E-2	1.29E-1	*2*	*79*	*0.005*	Homo sapiens
NR1H2 and NR1H3-mediated signaling	1	48	0.004	4.66E-2	1.39E-1	*1*	*60*	*0.004*	Homo sapiens
NCAM signaling for neurite out-growth	1	64	0.005	6.17E-2	1.39E-1	*1*	*23*	*0.002*	Homo sapiens
