# Supplementary material for: Proteomic Exploration of L1CAM+-Extracellular Vesicles from Plasma of Manifest and Prodromal Parkinson’s Disease
Source: Int J Mol Sci. 2025 Nov 28;26(23):11564. doi: 10.3390/ijms262311564 (PMC12692637; doi:10.3390/ijms262311564)
Supplement: Supplementary file 1 [file ijms-26-11564-s001.zip › Supplementary Tables S1 and S2 and Figures S1 and S2.pdf]

Supplementary Data

Using the R package *glmnet*, we generated in addition to the LASSO (alpha = 1) a RIDGE model (alpha = 0) and an ElasticNet model (alpha = 0.5). The following table gives the number of proteins retained by each method.

Method	Number Non zero features
Ridge	203
Lasso	12
ElasticNet	48

Supplementary Table S1. Number of non-zero features (retained proteins) the 3 methods: RIDGE, LASSO and ElasticNet.

The following table compares the Mean squared error (MSE), the Root Mean Squared Error (RMSE) and the Mean Absolute Error (MAE). Whereas the Lasso and Ridge are comparable, the ElasticNet produces values higher than 1. This means that the ElsaticNet model is not a better fit with the target value.

Method	MSE	RMSE	MAE
Ridge	0.372	0.610	0.503
Lasso	0.414	0.643	0.602
ElasticNet	0.709	0.842	0.815

Supplementary Table S2. MSE, RMSE and MAE of the 3 methods: RIDGE, LASSO and ElasticNet.

The following plot gives the ROC/AUC analysis of the 3 methods in the hold out test set. We observed that all 3 methods result in comparable AUCs.

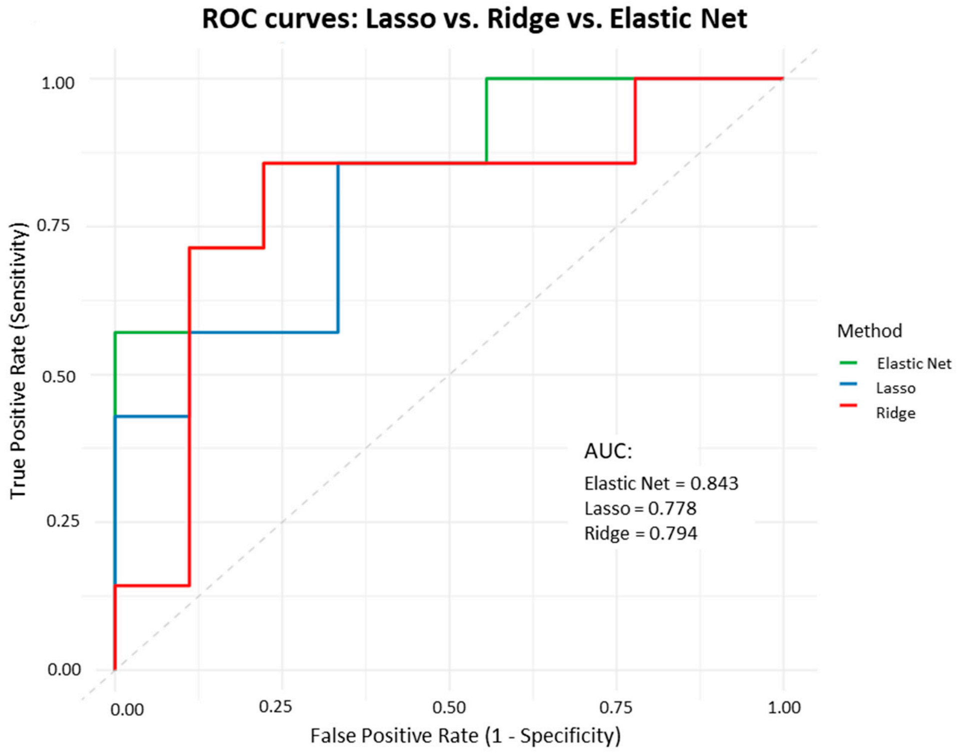

Supplementary Figure S1. ROC analysis of the 3 methods RIDGE, LASSO and ElasticNet.

The following plot compare the coefficients of the 12 proteins retained by LASSO to Ridge and ElasticNet. Many of the coefficients are of comparable magnitude between LASSO and ElasticNet, whereas the Ridge Method gives much smaller values for those 12 proteins.

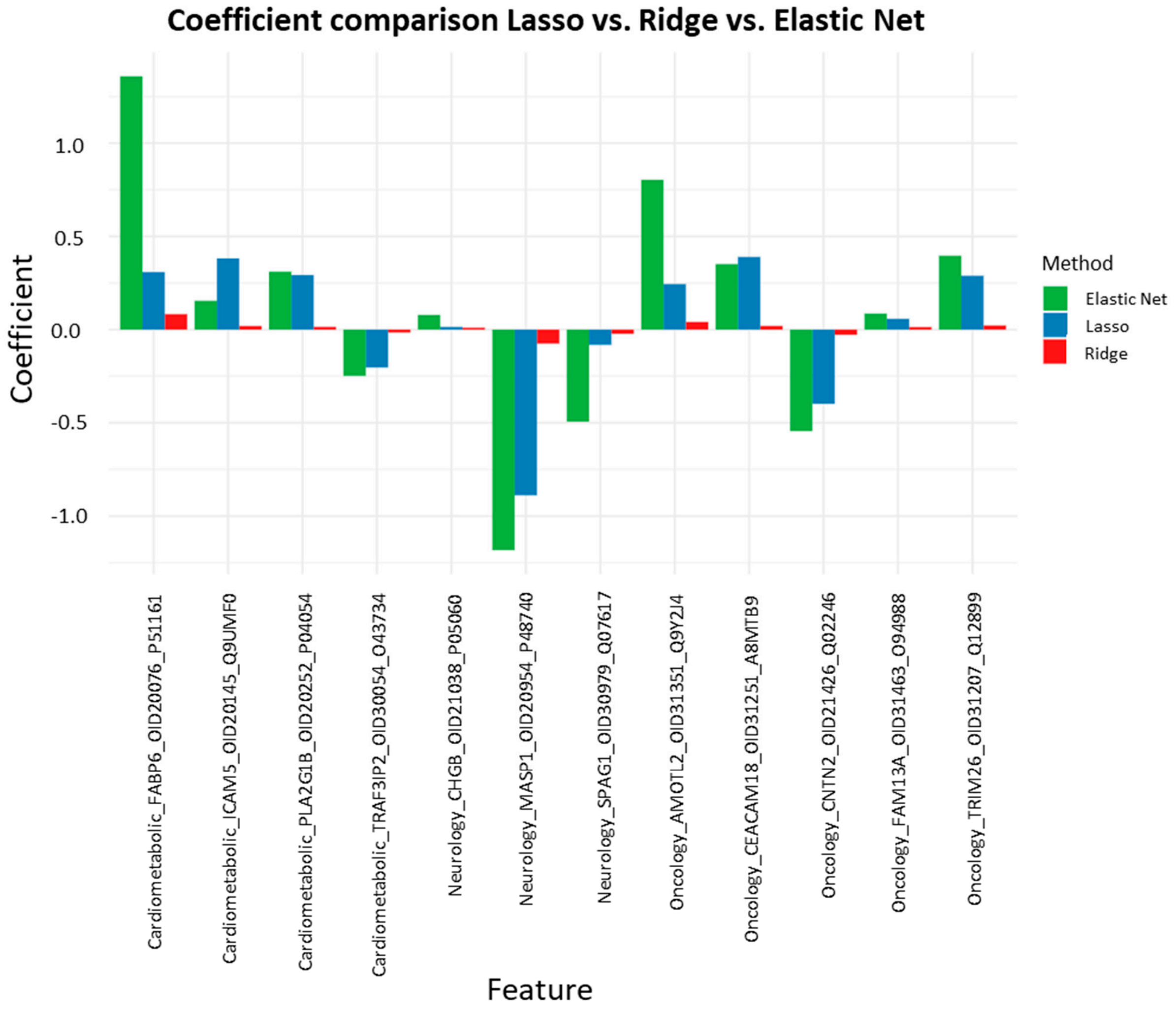

Supplementary Figure S2. The magnitude of the coefficients of the 12 proteins retained by LASSO is compared to its corresponding values in RIDGE and ElasticNet method.

The Ridge method is known for best handling collinearity; however, it keeps all features. Given the small sample size of the study we rather opted for the LASSO which best for feature selection, but may struggle with collinearity. The used LASSO model performance is, however, comparable to the Ridge model.

The ElasticNet is a balanced approach that handles both collinearity and performs feature selection. We do not opt for this model here, even though it generates a slightly higher AUC than the LASSO

(0.843 vs. 0.778) as for the ElasticNet model MSE, RMSE and MAE are higher than the other two methods and the number of selected features (48) is higher than LASSO (12).

Given the small sample size of the actual study, on comparing the prediction accuracy of the 3 methods, we see that the LASSO is a good choice for fitting the data at hand. This is in accordance with the *Occam's razor* principle for data analysis, which suggests choosing the most straightforward model that still achieves sufficient accuracy for the problem at hand.
