# Supplementary material for: Proteomic Exploration of L1CAM+-Extracellular Vesicles from Plasma of Manifest and Prodromal Parkinson’s Disease
Source: Int J Mol Sci. 2025 Nov 28;26(23):11564. doi: 10.3390/ijms262311564 (PMC12692637; doi:10.3390/ijms262311564)
Supplement: Supplementary file 1 [file ijms-26-11564-s001.zip › Supplementary Table S3.pdf]

Protein (Name as: Panel_Assay_OlinkID_UniProt)	HC	PD	FoldChange	Raw p.value	conf.low	conf.high	Regulation in PD	LASSO Model Coefficient Value
Cardiometabolic_TRAF3IP2_OID30054_O43734	-1.034	-2.366	1.332	0.008	0.368	2.296	Down regulated in PD	-0.889
Oncology_CNTN2_OID21426_Q02246	-6.882	-7.537	0.655	0.015	0.135	1.175	Down regulated in PD	-0.398
Neurology_MASP1_OID20954_P48740	-3.339	-3.486	0.147	0.081	-0.019	0.312	Down regulated in PD	-0.203
Neurology_SPAG1_OID30979_Q07617	-3.276	-3.708	0.432	0.156	-0.172	1.036	Down regulated in PD	-0.082
Oncology_AMOTL2_OID31351_Q9Y2J4	-0.004	0.406	-0.410	0.003	-0.674	-0.146	Up regulated in PD	0.014
Oncology_FAM13A_OID31463_O94988	-2.237	-0.949	-1.288	0.003	-2.123	-0.453	Up regulated in PD	0.058
Oncology_CEACAM18_OID31251_A8MTB9	-2.571	-0.945	-1.626	0.005	-2.723	-0.528	Up regulated in PD	0.244
Cardiometabolic_FABP6_OID20076_P51161	0.219	0.408	-0.189	0.015	-0.340	-0.039	Up regulated in PD	0.289
Oncology_TRIM26_OID31207_Q12899	-0.873	-0.155	-0.719	0.020	-1.317	-0.121	Up regulated in PD	0.293
Cardiometabolic_ICAM5_OID20145_Q9UMF0	-5.085	-4.228	-0.858	0.020	-1.573	-0.142	Up regulated in PD	0.309
Neurology_CHGB_OID21038_P05060	-4.269	-3.367	-0.902	0.044	-1.780	-0.024	Up regulated in PD	0.382
Cardiometabolic_PLA2G1B_OID20252_P04054	-2.690	-1.981	-0.709	0.055	-1.434	0.015	Up regulated in PD	0.390
