# Supplementary material for: Proteomic Exploration of L1CAM+-Extracellular Vesicles from Plasma of Manifest and Prodromal Parkinson’s Disease
Source: Int J Mol Sci. 2025 Nov 28;26(23):11564. doi: 10.3390/ijms262311564 (PMC12692637; doi:10.3390/ijms262311564)
Supplement: Supplementary file 1 [file ijms-26-11564-s001.zip › Supplementary Table S4.pdf]

Protein<br>(Name as: Panel_Assay_OlinkID_UniProt)	contrast	FoldChange	conf.low	conf.high	Tukey Anova adj. p-val
Oncology_CNTN2_OID21426_Q02246	HC - PD	0.655	0.068	1.242	0.025
Oncology_CNTN2_OID21426_Q02246	iRBD - PD	0.375	-0.212	0.963	0.281
Oncology_CNTN2_OID21426_Q02246	HC - iRBD	0.280	-0.308	0.867	0.490
Cardiometabolic_TRAF3IP2_OID30054_O43734	HC - PD	1.332	0.278	2.386	0.010
Cardiometabolic_TRAF3IP2_OID30054_O43734	iRBD - PD	1.203	0.149	2.256	0.022
Cardiometabolic_TRAF3IP2_OID30054_O43734	HC - iRBD	0.129	-0.925	1.183	0.953
Cardiometabolic_EXTL1_OID30114_Q92935	HC - PD	3.548	0.727	6.368	0.010
Cardiometabolic_EXTL1_OID30114_Q92935	HC - iRBD	2.316	-0.504	5.137	0.127
Cardiometabolic_EXTL1_OID30114_Q92935	iRBD - PD	1.231	-1.589	4.051	0.548
Neurology_DIPK1C_OID30932_Q0P6D2	HC - PD	0.370	0.080	0.661	0.009
Neurology_DIPK1C_OID30932_Q0P6D2	HC - iRBD	0.214	-0.077	0.504	0.188
Neurology_DIPK1C_OID30932_Q0P6D2	iRBD - PD	0.156	-0.134	0.447	0.403
Neurology_KIAA0319_OID31094_Q5VV43	HC - PD	1.410	0.010	2.811	0.048
Neurology_KIAA0319_OID31094_Q5VV43	iRBD - PD	1.124	-0.276	2.525	0.139
Neurology_KIAA0319_OID31094_Q5VV43	HC - iRBD	0.286	-1.115	1.687	0.876
Cardiometabolic_MMP7_OID20087_P09237	HC - PD	-0.841	-1.581	-0.101	0.022
Cardiometabolic_MMP7_OID20087_P09237	iRBD - PD	-0.679	-1.419	0.061	0.078
Cardiometabolic_MMP7_OID20087_P09237	HC - iRBD	-0.162	-0.902	0.578	0.858
Cardiometabolic_DNAJB8_OID20093_Q8NHS0	HC - PD	-0.133	-0.265	0.000	0.051
Cardiometabolic_DNAJB8_OID20093_Q8NHS0	iRBD - PD	-0.111	-0.244	0.022	0.117
Cardiometabolic_DNAJB8_OID20093_Q8NHS0	HC - iRBD	-0.021	-0.154	0.112	0.923
Cardiometabolic_SNX9_OID20135_Q9Y5X1	HC - PD	-0.715	-1.496	0.066	0.079
Cardiometabolic_SNX9_OID20135_Q9Y5X1	iRBD - PD	-0.707	-1.489	0.074	0.084
Cardiometabolic_SNX9_OID20135_Q9Y5X1	HC - iRBD	-0.008	-0.789	0.774	1.000
Cardiometabolic_SNAP23_OID20218_O00161	HC - PD	-0.910	-1.813	-0.007	0.048
Cardiometabolic_SNAP23_OID20218_O00161	iRBD - PD	-0.716	-1.619	0.187	0.146
Cardiometabolic_SNAP23_OID20218_O00161	HC - iRBD	-0.194	-1.097	0.708	0.863
Inflammation_NUB1_OID20510_Q9Y5A7	HC - PD	-0.971	-1.763	-0.180	0.012
Inflammation_NUB1_OID20510_Q9Y5A7	iRBD - PD	-0.608	-1.399	0.183	0.163
Inflammation_NUB1_OID20510_Q9Y5A7	HC - iRBD	-0.363	-1.155	0.428	0.515
Inflammation_PROK1_OID20543_P58294	HC - PD	-0.382	-0.738	-0.026	0.033
Inflammation_PROK1_OID20543_P58294	HC - iRBD	-0.263	-0.618	0.093	0.187
Inflammation_PROK1_OID20543_P58294	iRBD - PD	-0.119	-0.475	0.236	0.700
Inflammation_AXIN1_OID20582_O15169	HC - PD	-1.084	-2.124	-0.043	0.039
Inflammation_AXIN1_OID20582_O15169	iRBD - PD	-1.020	-2.060	0.021	0.056
Inflammation_AXIN1_OID20582_O15169	HC - iRBD	-0.064	-1.105	0.976	0.988
Inflammation_IL16_OID20633_Q14005	HC - PD	-0.953	-1.782	-0.124	0.021
Inflammation_IL16_OID20633_Q14005	iRBD - PD	-0.656	-1.485	0.173	0.147
Inflammation_IL16_OID20633_Q14005	HC - iRBD	-0.297	-1.126	0.532	0.667
Neurology_LY96_OID20945_Q9Y6Y9	HC - PD	-0.313	-0.573	-0.053	0.015
Neurology_LY96_OID20945_Q9Y6Y9	HC - iRBD	-0.184	-0.444	0.075	0.211
Neurology_LY96_OID20945_Q9Y6Y9	iRBD - PD	-0.128	-0.388	0.132	0.465
Neurology_DNMBP_OID20956_Q6XZF7	HC - PD	-0.867	-1.699	-0.034	0.039
Neurology_DNMBP_OID20956_Q6XZF7	iRBD - PD	-0.808	-1.640	0.025	0.059
Neurology_DNMBP_OID20956_Q6XZF7	HC - iRBD	-0.059	-0.891	0.773	0.984
Neurology_CXCL8_OID20997_P10145	HC - PD	-0.974	-1.956	0.007	0.052
Neurology_CXCL8_OID20997_P10145	iRBD - PD	-0.804	-1.786	0.178	0.129
Neurology_CXCL8_OID20997_P10145	HC - iRBD	-0.170	-1.152	0.812	0.909
Neurology_F11R_OID21151_Q9Y624	HC - PD	-0.575	-1.161	0.011	0.056
Neurology_F11R_OID21151_Q9Y624	iRBD - PD	-0.500	-1.086	0.086	0.109
Neurology_F11R_OID21151_Q9Y624	HC - iRBD	-0.075	-0.661	0.511	0.949
Oncology_VPS53_OID21281_Q5VIR6	HC - PD	-0.482	-0.973	0.009	0.056
Oncology_VPS53_OID21281_Q5VIR6	iRBD - PD	-0.467	-0.958	0.023	0.065
Oncology_VPS53_OID21281_Q5VIR6	HC - iRBD	-0.014	-0.505	0.477	0.997
Oncology_USO1_OID21367_O60763	HC - PD	-1.142	-2.208	-0.076	0.033
Oncology_USO1_OID21367_O60763	iRBD - PD	-0.774	-1.839	0.292	0.197
Oncology_USO1_OID21367_O60763	HC - iRBD	-0.368	-1.434	0.697	0.685
Oncology_SCLY_OID21425_Q96I15	HC - PD	-0.860	-1.623	-0.097	0.024
Oncology_SCLY_OID21425_Q96I15	HC - iRBD	-0.537	-1.300	0.226	0.216
Oncology_SCLY_OID21425_Q96I15	iRBD - PD	-0.323	-1.086	0.440	0.568
Oncology_CXCL8_OID21430_P10145	HC - PD	-1.083	-2.085	-0.082	0.031

Protein<br>(Name as: Panel_Assay_OlinkID_UniProt)	contrast	FoldChange	conf.low	conf.high	Tukey Anova adj. p-val
Oncology_CXCL8_OID21430_P10145	iRBD - PD	-0.706	-1.708	0.296	0.216
Oncology_CXCL8_OID21430_P10145	HC - iRBD	-0.378	-1.379	0.624	0.638
Cardiometabolic_PYY_OID30150_P10082	HC - PD	-3.047	-5.933	-0.161	0.036
Cardiometabolic_PYY_OID30150_P10082	iRBD - PD	-2.329	-5.215	0.557	0.136
Cardiometabolic_PYY_OID30150_P10082	HC - iRBD	-0.718	-3.604	2.168	0.821
Cardiometabolic_RNF5_OID30218_Q99942	HC - PD	-0.788	-1.580	0.005	0.052
Cardiometabolic_RNF5_OID30218_Q99942	iRBD - PD	-0.721	-1.513	0.071	0.081
Cardiometabolic_RNF5_OID30218_Q99942	HC - iRBD	-0.066	-0.858	0.726	0.978
Inflammation_DBN1_OID30549_Q16643	HC - PD	-0.960	-1.836	-0.085	0.028
Inflammation_DBN1_OID30549_Q16643	iRBD - PD	-0.859	-1.734	0.016	0.056
Inflammation_DBN1_OID30549_Q16643	HC - iRBD	-0.102	-0.977	0.774	0.958
Inflammation_APOB_OID30673_P04114	HC - PD	-0.840	-1.726	0.046	0.067
Inflammation_APOB_OID30673_P04114	iRBD - PD	-0.785	-1.671	0.101	0.092
Inflammation_APOB_OID30673_P04114	HC - iRBD	-0.055	-0.941	0.831	0.988
Oncology_TRIM26_OID31207_Q12899	HC - PD	-0.719	-1.359	-0.079	0.024
Oncology_TRIM26_OID31207_Q12899	iRBD - PD	-0.419	-1.059	0.221	0.265
Oncology_TRIM26_OID31207_Q12899	HC - iRBD	-0.300	-0.940	0.340	0.501
Oncology_CENPJ_OID31314_Q9HC77	HC - PD	-1.887	-3.618	-0.156	0.030
Oncology_CENPJ_OID31314_Q9HC77	HC - iRBD	-1.507	-3.238	0.224	0.100
Oncology_CENPJ_OID31314_Q9HC77	iRBD - PD	-0.380	-2.111	1.351	0.858
Oncology_AMOTL2_OID31351_Q9Y2J4	HC - PD	-0.410	-0.678	-0.143	0.001
Oncology_AMOTL2_OID31351_Q9Y2J4	iRBD - PD	-0.309	-0.576	-0.041	0.020
Oncology_AMOTL2_OID31351_Q9Y2J4	HC - iRBD	-0.102	-0.369	0.166	0.634
Oncology_LARP1_OID31372_Q6PKG0	HC - PD	-1.519	-2.693	-0.345	0.008
Oncology_LARP1_OID31372_Q6PKG0	iRBD - PD	-0.925	-2.099	0.249	0.149
Oncology_LARP1_OID31372_Q6PKG0	HC - iRBD	-0.594	-1.768	0.580	0.448
Oncology_USP25_OID31428_Q9UHP3	HC - PD	-0.755	-1.480	-0.030	0.040
Oncology_USP25_OID31428_Q9UHP3	iRBD - PD	-0.544	-1.270	0.181	0.177
Oncology_USP25_OID31428_Q9UHP3	HC - iRBD	-0.211	-0.936	0.514	0.764
