# Supplementary material for: Proteomic Exploration of L1CAM+-Extracellular Vesicles from Plasma of Manifest and Prodromal Parkinson’s Disease
Source: Int J Mol Sci. 2025 Nov 28;26(23):11564. doi: 10.3390/ijms262311564 (PMC12692637; doi:10.3390/ijms262311564)
Supplement: Supplementary file 1 [file ijms-26-11564-s001.zip › Supplementary Table S5.pdf]

Protein<br>(Name as: Panel_Assay_OlinkID_UniProt)	HC (N=20)	iRBD (N=20)	PD (N=20)	Non adj. anova<br>p value
Oncology_CNTN2_OID21426_Q02246				0.033
- Mean (SD)	-6.882 (0.636)	-7.162 (0.684)	-7.537 (0.956)	
- Median (Q1, Q3)	-6.825 (-7.305, -6.415)	-7.014 (-7.631, -6.670)	-7.445 (-8.220, -6.867)	
- Min - Max	-7.926 - -5.792	-8.717 - -6.106	-9.187 - -5.468	
- Missing	0	0	0	
Cardiometabolic_TRAF3IP2_OID30054_O43734				0.006
- Mean (SD)	-1.034 (0.899)	-1.163 (1.103)	-2.366 (1.931)	
- Median (Q1, Q3)	-0.883 (-1.142, -0.524)	-0.806 (-2.024, -0.378)	-1.905 (-2.492, -1.300)	
- Min - Max	-3.230 - 0.524	-3.287 - 0.109	-7.752 - -0.190	
- Missing	0	0	0	
Cardiometabolic_EXTL1_OID30114_Q92935				0.013
- Mean (SD)	-2.360 (2.353)	-4.677 (3.916)	-5.908 (4.508)	
- Median (Q1, Q3)	-1.831 (-2.784, -1.134)	-2.510 (-9.486, -2.197)	-5.086 (-10.291, -1.446)	
- Min - Max	-10.583 - 0.566	-11.251 - -1.235	-11.279 - -0.372	
- Missing	0	0	0	
Neurology_DIPK1C_OID30932_Q0P6D2				0.012
- Mean (SD)	-0.059 (0.396)	-0.272 (0.362)	-0.429 (0.386)	
- Median (Q1, Q3)	-0.065 (-0.246, 0.118)	-0.188 (-0.413, -0.093)	-0.453 (-0.676, -0.048)	
- Min - Max	-0.935 - 0.772	-1.047 - 0.289	-1.117 - 0.094	
- Missing	0	0	0	
Neurology_KIAA0319_OID31094_Q5VV43				0.045
- Mean (SD)	-6.198 (0.894)	-6.484 (1.615)	-7.608 (2.600)	
- Median (Q1, Q3)	-6.027 (-7.095, -5.414)	-6.197 (-6.607, -5.624)	-6.891 (-8.190, -5.777)	
- Min - Max	-8.105 - -4.918	-12.129 - -4.878	-13.171 - -4.907	
- Missing	0	0	0	
Cardiometabolic_DNAJB8_OID20093_Q8NHS0				0.043
- Mean (SD)	0.569 (0.184)	0.590 (0.182)	0.702 (0.157)	
- Median (Q1, Q3)	0.548 (0.455, 0.666)	0.613 (0.457, 0.732)	0.720 (0.644, 0.808)	
- Min - Max	0.208 - 0.966	0.223 - 0.863	0.224 - 0.914	
- Missing	0	0	0	
Cardiometabolic_MMP7_OID20087_P09237				0.020
- Mean (SD)	-6.202 (0.635)	-6.040 (0.661)	-5.361 (1.412)	
- Median (Q1, Q3)	-6.321 (-6.571, -5.715)	-6.090 (-6.551, -5.697)	-5.773 (-6.203, -5.366)	
- Min - Max	-7.533 - -5.213	-7.048 - -4.466	-6.910 - -1.266	
- Missing	0	0	0	
Cardiometabolic_PYY_OID30150_P10082				0.036
- Mean (SD)	-5.962 (3.687)	-5.244 (3.877)	-2.915 (3.811)	
- Median (Q1, Q3)	-7.739 (-8.627, -4.038)	-7.515 (-8.426, -0.952)	-0.953 (-7.107, -0.026)	
- Min - Max	-9.041 - 1.048	-9.083 - 1.014	-8.863 - 0.858	
- Missing	0	0	0	
Cardiometabolic_RNF5_OID30218_Q99942				0.036
- Mean (SD)	4.803 (1.207)	4.870 (1.075)	5.591 (0.800)	
- Median (Q1, Q3)	4.683 (3.849, 5.468)	4.851 (4.176, 5.800)	5.742 (5.317, 6.073)	
- Min - Max	3.013 - 7.526	2.388 - 6.305	3.304 - 6.535	
- Missing	0	0	0	
Cardiometabolic_SNAP23_OID20218_O00161				0.045
- Mean (SD)	6.278 (1.387)	6.472 (1.292)	7.188 (0.793)	
- Median (Q1, Q3)	6.335 (5.490, 7.243)	6.595 (5.819, 7.335)	7.376 (6.653, 7.811)	
- Min - Max	3.601 - 8.673	2.773 - 8.030	4.870 - 8.082	
- Missing	0	0	0	
Cardiometabolic_SNX9_OID20135_Q9Y5X1				0.048
- Mean (SD)	1.361 (1.090)	1.369 (0.919)	2.076 (1.063)	
- Median (Q1, Q3)	1.141 (0.617, 2.179)	1.259 (0.900, 1.840)	2.046 (1.310, 2.622)	
- Min - Max	-0.233 - 3.765	-0.319 - 3.232	0.103 - 4.562	
- Missing	0	0	0	
Inflammation_APOB_OID30673_P04114				0.046
- Mean (SD)	3.040 (0.660)	3.095 (1.207)	3.880 (1.475)	
- Median (Q1, Q3)	2.859 (2.605, 3.345)	2.961 (2.280, 3.560)	3.548 (3.208, 4.288)	
- Min - Max	1.916 - 4.301	1.524 - 6.518	1.946 - 8.626	
- Missing	0	0	0	

**Protein** (Name as: Panel_Assay_OlinkID_UniProt)	HC (N=20)	iRBD (N=20)	PD (N=20)	Non adj. anova p value
Inflammation_AXIN1_OID20582_O15169				0.025
- Mean (SD)	0.795 (1.053)	0.860 (1.267)	1.879 (1.701)	
- Median (Q1, Q3)	0.604 (0.105, 1.354)	0.581 (0.171, 1.554)	1.432 (0.742, 2.357)	
- Min - Max	-0.698 - 3.582	-1.979 - 3.405	-0.158 - 5.926	
- Missing	0	0	0	
Inflammation_DBN1_OID30549_Q16643				0.020
- Mean (SD)	-0.516 (1.356)	-0.414 (0.967)	0.444 (1.093)	
- Median (Q1, Q3)	-0.460 (-0.880, 0.059)	-0.566 (-0.905, 0.171)	0.345 (-0.235, 0.683)	
- Min - Max	-4.541 - 1.843	-2.365 - 1.657	-1.305 - 2.840	
- Missing	0	0	0	
Inflammation_IL16_OID20633_Q14005				0.024
- Mean (SD)	-5.512 (0.736)	-5.215 (1.203)	-4.559 (1.253)	
- Median (Q1, Q3)	-5.583 (-5.860, -5.019)	-5.200 (-5.890, -4.804)	-4.795 (-5.303, -4.068)	
- Min - Max	-7.215 - -3.580	-7.128 - -2.036	-6.172 - -0.818	
- Missing	0	0	0	
Inflammation_NUB1_OID20510_Q9Y5A7				0.016
- Mean (SD)	-1.314 (1.334)	-0.950 (0.855)	-0.342 (0.856)	
- Median (Q1, Q3)	-1.575 (-2.105, -0.640)	-1.001 (-1.525, -0.435)	-0.365 (-0.922, 0.311)	
- Min - Max	-3.596 - 2.307	-2.309 - 0.552	-1.889 - 1.632	
- Missing	0	0	0	
Inflammation_PROK1_OID20543_P58294				0.037
- Mean (SD)	-4.718 (0.547)	-4.456 (0.427)	-4.337 (0.417)	
- Median (Q1, Q3)	-4.752 (-5.135, -4.363)	-4.530 (-4.682, -4.072)	-4.417 (-4.654, -4.197)	
- Min - Max	-5.492 - -3.656	-5.472 - -3.816	-4.839 - -3.218	
- Missing	0	0	0	
Neurology_CXCL8_OID20997_P10145				0.046
- Mean (SD)	-5.006 (1.004)	-4.836 (1.418)	-4.032 (1.406)	
- Median (Q1, Q3)	-5.000 (-5.711, -4.414)	-5.303 (-5.856, -4.209)	-4.238 (-4.739, -3.598)	
- Min - Max	-6.866 - -3.108	-6.262 - -0.945	-5.902 - -0.462	
- Missing	0	0	0	
Neurology_DNMBP_OID20956_Q6XZF7				0.025
- Mean (SD)	-3.088 (0.989)	-3.029 (0.878)	-2.221 (1.356)	
- Median (Q1, Q3)	-3.275 (-3.792, -2.569)	-3.082 (-3.394, -2.585)	-2.483 (-3.036, -1.875)	
- Min - Max	-4.849 - -0.373	-4.639 - -1.120	-4.263 - 1.585	
- Missing	0	0	0	
Neurology_F11R_OID21151_Q9Y624				0.044
- Mean (SD)	6.422 (0.856)	6.497 (0.919)	6.997 (0.449)	
- Median (Q1, Q3)	6.494 (5.960, 7.094)	6.649 (6.179, 7.165)	7.121 (6.759, 7.307)	
- Min - Max	4.524 - 7.455	3.686 - 7.370	5.646 - 7.519	
- Missing	0	0	0	
Neurology_LY96_OID20945_Q9Y6Y9				0.019
- Mean (SD)	-3.606 (0.253)	-3.422 (0.360)	-3.294 (0.396)	
- Median (Q1, Q3)	-3.640 (-3.764, -3.467)	-3.387 (-3.620, -3.224)	-3.336 (-3.534, -3.054)	
- Min - Max	-4.063 - -3.128	-4.344 - -2.765	-4.147 - -2.632	
- Missing	0	0	0	
Oncology_AMOTL2_OID31351_Q9Y2J4				0.001
- Mean (SD)	-0.004 (0.313)	0.097 (0.176)	0.406 (0.492)	
- Median (Q1, Q3)	-0.051 (-0.185, 0.014)	0.075 (0.008, 0.202)	0.280 (0.134, 0.454)	
- Min - Max	-0.356 - 1.035	-0.275 - 0.487	-0.117 - 1.935	
- Missing	0	0	0	
Oncology_CENPJ_OID31314_Q9HC77				0.027
- Mean (SD)	-1.452 (3.055)	0.055 (0.917)	0.435 (2.311)	
- Median (Q1, Q3)	-0.584 (-1.039, 0.495)	-0.087 (-0.599, 0.813)	0.764 (-0.318, 1.529)	
- Min - Max	-7.913 - 1.241	-1.700 - 1.704	-7.901 - 3.238	
- Missing	0	0	0	
Oncology_CXCL8_OID21430_P10145				0.037
- Mean (SD)	-4.907 (1.107)	-4.530 (1.355)	-3.824 (1.462)	
- Median (Q1, Q3)	-4.699 (-5.798, -4.230)	-5.001 (-5.360, -3.988)	-4.072 (-4.386, -3.125)	
- Min - Max	-6.371 - -2.669	-6.197 - -0.669	-6.105 - -0.191	
- Missing	0	0	0	
Oncology_LARP1_OID31372_Q6PKG0				0.011

**Protein**<br>**(Name as: Panel_Assay_OlinkID_UniProt)**	**HC (N=20)**	**iRBD (N=20)**	**PD (N=20)**	**Non adj. anova**<br>**p value**
- Mean (SD)	-3.509 (1.845)	-2.915 (1.369)	-1.990 (1.365)	
- Median (Q1, Q3)	-3.210 (-4.415, -2.733)	-3.043 (-3.715, -2.057)	-1.983 (-2.862, -1.383)	
- Min - Max	-9.145 - 0.273	-5.397 - 0.380	-4.465 - 0.828	
- Missing	0	0	0	
Oncology_SCLY_OID21425_Q96I15				0.029
- Mean (SD)	-6.699 (1.310)	-6.161 (0.659)	-5.838 (0.930)	
- Median (Q1, Q3)	-6.581 (-7.276, -5.818)	-6.326 (-6.530, -5.593)	-5.830 (-6.142, -5.349)	
- Min - Max	-9.537 - -3.915	-7.559 - -5.046	-7.984 - -4.215	
- Missing	0	0	0	
Oncology_TRIM26_OID31207_Q12899				0.031
- Mean (SD)	-0.873 (1.071)	-0.573 (0.614)	-0.155 (0.774)	
- Median (Q1, Q3)	-0.851 (-1.498, -0.046)	-0.512 (-0.899, -0.237)	-0.276 (-0.588, 0.322)	
- Min - Max	-3.141 - 0.896	-1.944 - 0.584	-1.502 - 1.212	
- Missing	0	0	0	
Oncology_USO1_OID21367_O60763				0.038
- Mean (SD)	-0.716 (1.747)	-0.347 (1.077)	0.426 (1.293)	
- Median (Q1, Q3)	-0.983 (-2.087, 0.637)	0.034 (-0.982, 0.338)	0.446 (-0.687, 1.398)	
- Min - Max	-3.074 - 3.082	-3.022 - 1.350	-1.672 - 2.553	
- Missing	0	0	0	
Oncology_USP25_OID31428_Q9UHP3				0.042
- Mean (SD)	-1.410 (1.087)	-1.199 (0.679)	-0.655 (1.040)	
- Median (Q1, Q3)	-1.684 (-2.072, -0.808)	-1.246 (-1.581, -0.775)	-0.538 (-1.490, 0.143)	
- Min - Max	-3.021 - 1.366	-2.454 - 0.267	-2.916 - 1.144	
- Missing	0	0	0	
Oncology_VPS53_OID21281_Q5VIR6				0.033
- Mean (SD)	0.323 (0.644)	0.337 (0.513)	0.805 (0.755)	
- Median (Q1, Q3)	0.149 (0.042, 0.454)	0.286 (-0.045, 0.576)	0.538 (0.314, 1.290)	
- Min - Max	-0.504 - 2.522	-0.332 - 1.830	-0.039 - 2.442	
- Missing	0	0	0	
